# Predictive factors of postoperative fentanyl consumption in patients with inflammatory bowel disease: a retrospective cohort study

**DOI:** 10.1186/s12871-022-01606-8

**Published:** 2022-03-11

**Authors:** Sayaka Tsuboi, Kazumi Kubota, Takahiro Mihara, Masataka Taguri, Gaku Inagawa, Takahisa Goto

**Affiliations:** 1 Department of Biostatistics, Yokohama City University School of Medicine, 3-9 Fukuura, Kanazawa-ku, Yokohama, Kanagawa 236-0004 Japan; 2 Department of Anesthesia, Yokohama Municipal Citizen’s Hospital, 1-1 Mitsuzawa Nishimachi, Kanagawa-ku, Yokohama, Kanagawa 221-0855 Japan; 3 Department of Healthcare Information Management, The University of Tokyo Hospital, 7-3-1 Hongo, Bunkyo-ku, Tokyo, 113-8655 Japan; 4 Department of Health Data Science, Yokohama City University Graduate School of Data Science, 22-2 Seto, Kanazawa-ku, Yokohama, Kanagawa 236-0027 Japan; 5 Department of Anesthesiology, Yokohama City University School of Medicine, 3-9 Fukuura, Kanazawa-ku, Yokohama, Kanagawa 236-0004 Japan; 6 Department of Data Science, Yokohama City University Graduate School of Data Science, 22-2 Seto, Kanazawa-ku, Yokohama, Kanagawa 236-0027 Japan

**Keywords:** Inflammatory bowel disease, Fentanyl, Analgesia, Patient-controlled analgesia, Opioid

## Abstract

**Background:**

Patients with inflammatory bowel disease (IBD), including Crohn’s disease and ulcerative colitis, might present difficulties in achieving postoperative analgesia. Prior studies have suggested that patients with IBD undergoing major abdominal surgery require higher doses of perioperative opioids than do patients without IBD. Considering patients with IBD potentially require high-dose opioids, identifying those requiring higher opioid doses will allow clinicians to optimize the perioperative opioid dose and avoid insufficient pain management or complications of opioid overdose. Therefore, we conducted this study to identify predictive factors that might influence postoperative opioid consumption in patients with IBD.

**Methods:**

This single-center, historical cohort study reviewed the medical records of all patients admitted to the IBD center of our institution for surgery and who used intravenous fentanyl patient-controlled analgesia (PCA) after open abdominal surgery between June 2013 and April 2017. Ultimately, 179 patients were enrolled in the analysis. Variables expected to influence and/or represent pain, analgesia, inflammation, disease condition, and extent of surgery were selected as potential explanatory variables for predicting postoperative opioid consumption. Multivariable linear regression analysis was used to examine the effect of independent variables on postoperative fentanyl consumption.

**Results:**

Of the nine predictive variables selected using the stepwise-selection method, eight were significant. Intraoperative fentanyl consumption, current smoking, ulcerative colitis, administration of biologics during the month before surgery, and the use of supplementary analgesics had a significant increasing effect on postoperative fentanyl consumption, whereas droperidol concentration in the PCA solution, age, and diabetes mellitus had a significant decreasing effect. Preoperative use of opioids was a non-significant variable. The adjusted coefficient of determination was 0.302.

**Conclusions:**

Intraoperative fentanyl consumption, current smoking, ulcerative colitis, administration of biologics during the month before surgery, and the use of supplementary analgesics had a significant increasing effect, whereas droperidol concentration in the PCA solution, age, and diabetes mellitus had a significant decreasing effect on postoperative fentanyl consumption. These factors should be considered when adopting postoperative intravenous fentanyl PCA administration for patients with IBD.

**Trial registration:**

Registry: UMIN Clinical Trials Registry.

Clinical Trial Number: UMIN000031198.

Date of registration: February 8, 2018.

## Background

Inflammatory bowel disease (IBD), which includes Crohn’s disease (CD) and ulcerative colitis (UC), is a chronic inflammatory disorder of the bowel whose etiology is still not fully understood. Patients with IBD often experience symptoms such as abdominal pain, diarrhea, and rectal bleeding. Surgical and medical treatments play a major role in managing IBD, and because many patients experience remitting and relapsing phases of inflammation, repeated surgery is common.

Patients with IBD undergoing surgery present difficulties in achieving postoperative analgesia. They often have multiple risk factors for postoperative pain: they are younger than other patients requiring abdominal surgeries, often use analgesics preoperatively, and occasionally present with an altered coagulation status (e.g., prolonged prothrombin time and/or activated partial thromboplastin time) [1], which make it difficult for anesthesiologists to administer epidural analgesia. In our institution, epidural analgesia is the first choice for postoperative analgesia in laparotomy for patients with IBD. For patients with an altered coagulation status, intravenous fentanyl is administered instead. Prior studies have suggested that patients with IBD undergoing major abdominal surgery require higher doses of perioperative opioids than do patients without IBD undergoing similar abdominal surgery [2–5]. In a previous study, Fleyfel et al. compared perioperative opioid consumption between the acute inflammatory phase and remission phase in the same patient and concluded that the inflammatory status affected opioid requirements in patients with UC undergoing surgery [6].

IBD itself may be a risk factor for an increased requirement of perioperative opioids, but other factors, such as the inflammatory status, may function as additional predictive factors for identifying patients who need high-dose opioids. Considering that patients with IBD potentially require high-dose opioids, detecting those who require higher opioid doses will allow clinicians to optimize the perioperative opioid dose and avoid insufficient pain management or complications of opioid overdose.

The aim of this study was to identify predictive factors that might influence postoperative opioid consumption in patients with IBD, with an emphasis on the preoperative inflammatory status. To be clinically practical, the candidate predictive factors were all selected from routine preoperative information.

## Methods

This single-center, historical cohort study was conducted at the Yokohama Municipal Citizen’s Hospital (YMCH), Yokohama, Kanagawa, Japan. The ethics board of the YMCH approved this study (approval No. 17–09-01) and waived the need for informed consent. This trial was registered at the University Hospital Medical Information Network Clinical Trials Registry (registration number, UMIN000031198; principal investigator, Sayaka Tsuboi; date of registration, February 8, 2018). This manuscript adheres to the applicable STROBE guidelines.

The perioperative medical records of all patients admitted for surgery to the IBD center of the YMCH and who used intravenous fentanyl patient-controlled analgesia (PCA) after open abdominal surgery between June 2013 and April 2017 were reviewed retrospectively. Clinical data were collected from the patients’ medical, surgical, anesthesia, and PCA records. The exclusion criteria were as follows: incomplete PCA records, opting out, inability to use the PCA instrument unaided, hepatic failure, renal failure, and blood disorders. We also excluded patients who used epidural analgesia for postoperative pain control, which was the first choice of analgesia in our institution. In general, coagulopathy, personal preference, and neurological disorders are reasons for not choosing epidural anesthesia, but in this study, all patients who chose intravenous fentanyl were included, regardless of the reason.

The data of interest were patient demographics, preoperative blood test data, disease characteristics, operative details, anesthetic details, and postoperative outcomes. Among these datasets, variables expected to influence and/or represent pain, analgesia, inflammation, disease condition, and extent of surgery were widely selected as potential explanatory variables, based on the recommendations of prior studies [7–10] or as the clinical basis for predicting postoperative opioid consumption. Patient demographics included age, sex, height, body weight, body mass index, current smoking status, and comorbidities. The preoperative blood test data included white blood cell (WBC) count, platelet count, neutrophil-to-lymphocyte ratio (NLR), platelet-to-lymphocyte ratio (PLR), C-reactive protein (CRP) level, hemoglobin level, 60-min erythrocyte sedimentation rate (ESR), estimated glomerular filtration rate (revised equations for the Japanese population) [11], fibrinogen level, and serum albumin level. The latest preoperative blood test data, no older than 60 days before surgery, were analyzed. Disease characteristics included diagnosis, disease duration, preoperative medical treatment (use of steroids, 5-aminosalicylic acids, immunosuppressants, biologics, or apheresis during the month before surgery), preoperative use of opioids or non-opioid analgesics, and a history of laparotomy. Operative and anesthetic details included operative time, presence of a supraumbilical incision, estimated blood loss, blood transfusion, type of anesthesia (volatile or intravenous), addition of peripheral nerve block (transversus abdominis plane block and/or quadratus lumborum block), intraoperative fentanyl dosage, use of antiemetics (droperidol and/or dexamethasone) during surgery, droperidol concentration in the PCA solution, and the use of supplementary analgesics other than fentanyl or remifentanil during anesthesia. We also investigated postoperative adverse outcomes, including postoperative nausea and vomiting (PONV), severe respiratory depression, 30-day mortality, and uncontrolled pain necessitating an unscheduled intervention by a doctor.

The primary outcome was fentanyl consumption per kilogram of body weight during the first 24 postoperative hours. The record of hourly fentanyl consumption retrieved from the CADD-Legacy PCA Pump Model 6300 (Smiths Medical MD, Inc., St. Paul, MN, USA) was investigated. All patients received continuous infusion of fentanyl as set by their anesthesiologist and additional bolus doses as requested by the patient. The start time of PCA was expected to vary among patients; therefore, we defined “postoperative consumption” as the amount of fentanyl administered after the patient left the operating room (which was almost the same as “after leaving the post-anesthesia care unit,” since our institution did not have a post-anesthesia care unit). Any dose administered before this was counted as intraoperative fentanyl consumption.

To obtain a correlation coefficient of 0.3, with an alpha error of 0.05 and a statistical power of 0.85, we calculated that 96 patients were needed for analysis (G*Power Version 3.1.9.2) [12]. Considering that approximately 50–60 patients with IBD per year used intravenous fentanyl PCA at our institution, approximately 200 patients were expected to be investigated for inclusion, which seemed sufficient even after excluding unsuitable cases.

### Statistical analysis

Continuous variables were expressed as mean (standard deviation). Multivariable linear regression analysis was used to examine the effect of independent variables on postoperative fentanyl consumption (mcg/kg/day). The dependent variable was log-transformed. Model selection was based on a stepwise-selection method using *P* < 0.05 and *P* > 0.05 as the inclusion and exclusion criteria, respectively. Multicollinearity of variables was assessed using variance inflation factor (VIF) analysis. A VIF value below 5 was considered acceptable. The listwise deletion method was used for handling missing data. All statistical analyses were performed using JMP Pro version 15.0.0 (SAS Institute Inc., Cary, NC, USA). Statistical significance was set at *P* < 0.05.

## Results

**Fig. 1:**
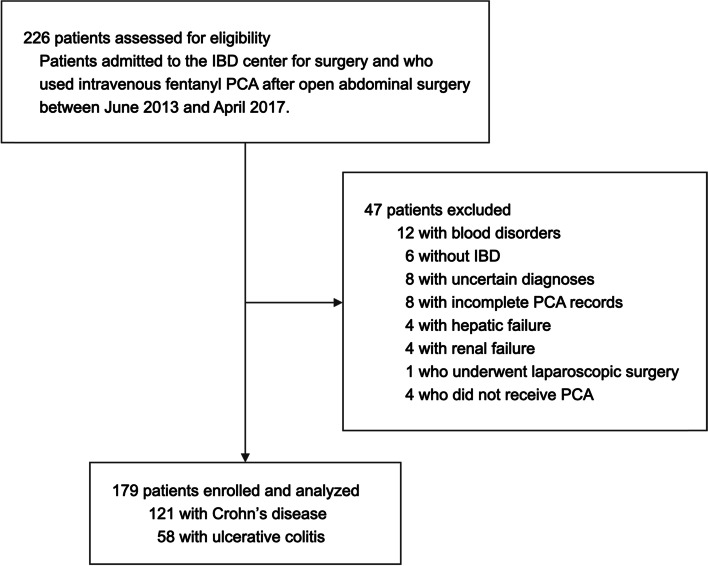
Flow diagram illustrating patient selection. IBD, inflammatory bowel syndrome; PCA, patient-controlled analgesia

Among the 226 patients who were assessed for eligibility, 179 were enrolled in the analysis (Fig. 1). Patient demographics and baseline characteristics are shown in Table 1. Coagulopathy, anemia, and hypoalbuminemia were the most common comorbidities. In total, 138 patients received nerve blocks as part of their multimodal analgesia. All nerve blocks were administered preoperatively under general anesthesia, and all of those were single-shot blocks. Missing data were found in 13, 5, 1, and 1 patient(s) for 60-min ESR, fibrinogen level, NLR, and PLR respectively.

**Table 1:** Patient demographics and baseline characteristics

	***n*** = 179
Age, mean (SD), y	37.3 (13.3)
Male, n (%)	130 (72.6)
Body mass index, mean (SD), kg/m^2^	18.8 (3.06)
Physical status, n (%)	
ASA 1	3 (1.7)
ASA 2	167 (93.3)
ASA 3	9 (5.0)
Comorbidities, n (%)	
Coagulopathy	149 (83.2)
Anemia	146 (81.6)
Hypoalbuminemia	120 (67.0)
Bronchial asthma	7 (3.9)
Diabetes mellitus	6 (3.4)
Hypertension	6 (3.4)
Ischemic heart disease	5 (2.8)
Cerebrovascular disease	2 (1.1)
Current smoking, n (%)	23 (12.8)
Nerve blocks, n (%)	
Preoperative TAP block	88 (49.2)
Preoperative QLB	48 (26.8)
Preoperative TAP & QLB	2 (1.1)
Operative time, mean (SD), min	243.8 (87.2)
Intraoperative fentanyl consumption, mean (SD), mcg	12.6 (4.2)
Postoperative fentanyl consumption, mean (SD), mcg/kg/day	30.7 (14.1)

*Abbreviations*: *ASA* American Society of Anesthesiologists, *QLB* Quadratus lumborum block, *SD* Standard deviation, *TAP* Transversus abdominis plane

### Primary outcome

**Fig. 2:**
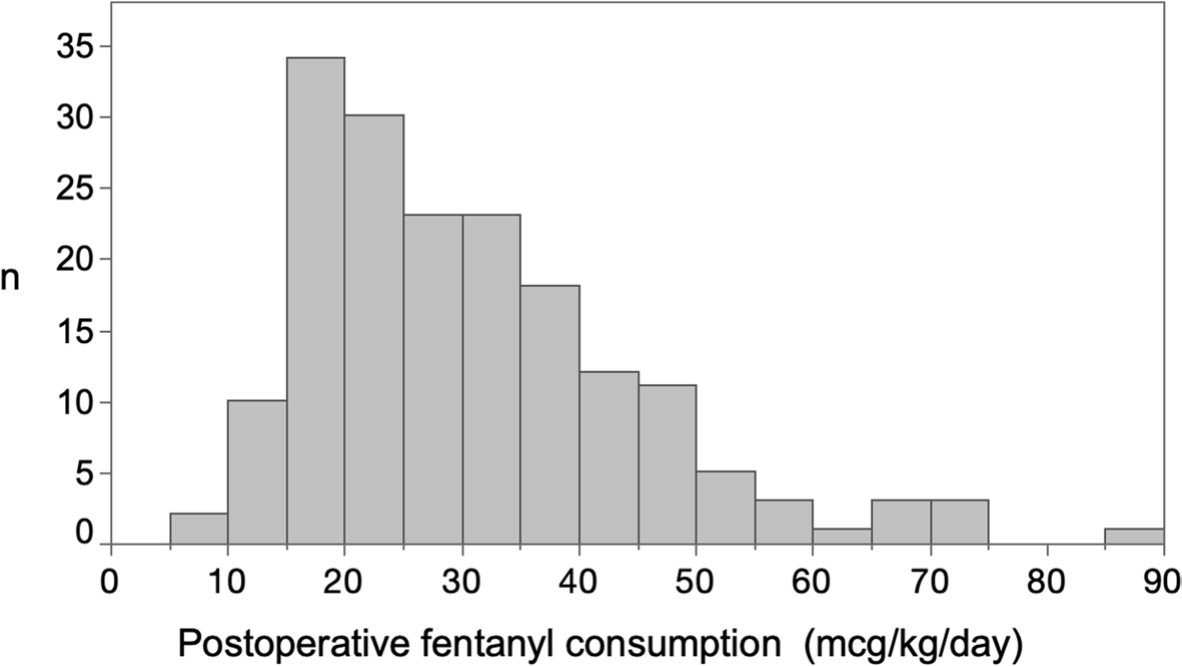
Histogram of fentanyl consumption during the first 24 postoperative hours (mcg/kg/day)

The mean fentanyl consumption in the first 24 postoperative hours was 30.7 (14.1) mcg/kg/day. The distribution of postoperative fentanyl consumption is shown in Fig. 2. Univariable analysis of the potential explanatory variables are shown in Tables 2 and 3. Nine predictive variables were selected after stepwise selection (Table 4). Eight of these variables (droperidol concentration in the PCA solution, intraoperative fentanyl consumption, age, diabetes mellitus, current smoking, diagnosis of IBD (UC or CD), use of supplementary analgesics during anesthesia, and administration of biologics during the month before surgery) showed a statistically significant influence on postoperative fentanyl consumption (Table 4). In contrast, preoperative use of opioids was the non-significant variable incorporated into the multivariable linear regression model. The adjusted coefficient of determination was 0.302. The partial regression coefficient, 95% confidence interval, standardized beta, and *P*-value of each explanatory variable for the multivariable linear regression model are shown in Table 4.

**Table 2:** Univariable analysis of postoperative fentanyl consumption (correlation analysis)

	Correlation coefficient	95% CI (lower, upper)	***P***-value
Age	−0.199	−0.336, − 0.054	0.008
Height	0.021	−0.126, 0.168	0.782
BMI	−0.158	−0.298, − 0.012	0.035
Operative time	− 0.041	− 0.186, 0.107	0.589
Blood loss	−0.097	−0.241, 0.050	0.194
Blood transfusion	0.004	−0.143, 0.151	0.957
Intraoperative fentanyl	−0.280	0.138, 0.410	0.0001
Intraoperative droperidol	−0.088	−0.232, 0.059	0.241
Droperidol concentration in the PCA solution	−0.217	− 0.353, − 0.073	0.004
Intraoperative dexamethasone	− 0.036	− 0.182, 0.111	0.632
WBC	0.021	−0.126, 0.167	0.777
Platelet count	0.153	0.007, 0.293	0.040
Hemoglobin	−0.096	−0.239. 0.051	0.201
NLR^a^	−0.020	−0.166, 0.128	0.792
PLR^a^	−0.139	−0.280, 0.009	0.065
CRP	0.124	−0.023, 0.266	0.099
60-min ESR^b^	0.090	−0.063, 0.239	0.248
eGFR [11]	0.205	0.060, 0.341	0.006
Fibrinogen^c^	0.085	−0.064, 0.231	0.263
Serum albumin	−0.060	−0.204, 0.088	0.429
Disease duration	−0.166	−0.305, − 0.020	0.026
Past laparotomy	−0.074	− 0.218, 0.074	0.325

^a^1 missing data point deleted

^b^13 missing data points deleted

^c^5 missing data points deleted

*Abbreviations*: *BMI* Body mass index, *CI* Confidence interval, *CRP* C-reactive protein, *eGFR* Estimated glomerular filtration rate, *ESR* Erythrocyte sedimentation rate, *NLR* Neutrophil-to-lymphocyte ratio, *PCA* Patient-controlled analgesia, *PLR* Platelet-to-lymphocyte ratio, *WBC* White blood cell count

**Table 3:** Univariable analysis of postoperative fentanyl consumption (Welch’s t-test)

	Fentanyl consumption, mean (SD), ln [mcg/kg/day]	95% CI (lower, upper)	***P***-value
Sex			
Male, *n* = 130	3.34 (0.443)	3.27, 3.42	0.323
Female, *n* = 49	3.27 (0.464)	3.13, 3.40	
Diagnosis			
Ulcerative colitis, *n* = 58	3.46 (0.423)	3.35, 3.58	0.003
Crohn’s disease, *n* = 121	3.26 (0.447)	3.18, 3.34	
Current smoking			
Yes, *n* = 23	3.57 (0.431)	3.39, 3.76	0.006
No, *n* = 156	3.29 (0.441)	3.22, 3.36	
Coagulopathy			
Yes, *n* = 149	3.34 (0.427)	3.28, 3.41	0.246
No, *n* = 30	3.22 (0.540)	3.02, 3.42	
Bronchial asthma			
Yes, *n* = 7	3.58 (0.184)	3.41, 3.75	0.007
No, *n* = 172	3.31 (0.454)	3.24, 3.38	
Diabetes mellitus			
Yes, *n* = 6	2.87 (0.346)	2.51, 3.24	0.020
No, *n* = 173	3.34 (0.445)	3.27, 3.41	
Hypertension			
Yes, *n* = 6	3.27 (0.560)	2.68, 3.85	0.809
No, *n* = 173	3.33 (0.446)	3.26, 3.39	
Ischemic heart disease			
Yes, *n* = 2	3.32 (0.290)	0.71, 5.92	0.973
No, *n* = 177	3.32 (0.451)	3.26, 3.39	
Cerebrovascular disease			
Yes, *n* = 2	2.87 (0.007)	2.80, 2.93	< 0.001
No, *n* = 177	3.33 (0.449)	3.26, 3.40	
Anesthesia maintenance			
Volatile, *n* = 130	3.37 (0.438)	3.29, 3.44	0.042
Intravenous, *n* = 49	3.21 (0.463)	3.08, 3.34	
Peripheral nerve block			
Yes, *n* = 138	3.32 (0.442)	3.24, 3.39	0.658
No, *n* = 41	3.35 (0.475)	3.20, 3.50	
Supraumbilical extension of the incision			
Yes, *n* = 59	3.22 (0.460)	3.10, 3.34	0.036
No, *n* = 120	3.37 (0.436)	3.30, 3.45	
Preoperative use of non-opioid analgesics			
Yes, *n* = 70	3.41 (0.468)	3.30, 3.52	0.050
No, *n* = 109	3.27 (0.430)	3.19, 3.35	
Preoperative use of opioids			
Yes, *n* = 20	3.53 (0.488)	3.30, 3.75	0.059
No, *n* = 159	3.30 (0.439)	3.23, 3.37	
Supplementary use of analgesics			
Yes, *n* = 105	3.34 (0.442)	3.26, 3.43	0.432
No, *n* = 74	3.29 (0.460)	3.19, 3.40	
Medical treatment during the month before surgery			
Steroids			
Yes, *n* = 46	3.40 (0.383)	3.29, 3.51	0.145
No, *n* = 133	3.30 (0.468)	3.22, 3.38	
5-Aminosalicylic acids			
Yes, *n* = 127	3.31 (0.443)	3.23, 3.39	0.544
No, *n* = 52	3.36 (0.466)	3.23, 3.49	
Immunosuppressants			
Yes, *n* = 52	3.37 (0.424)	3.25, 3.49	0.373
No, *n* = 127	3.31 (0.459)	3.22, 3.39	
Biologics			
Yes, *n* = 33	3.58 (0.459)	3.42, 3.74	< 0.001
No, *n* = 146	3.27 (0.427)	3.20, 3.34	
Apheresis			
Yes, *n* = 17	3.39 (0.364)	3.20, 3.58	0.442
No, *n* = 162	3.32 (0.457)	3.25, 3.39	

*Abbreviations*: *ASA* American Society of Anesthesiologists, *CI* Confidence interval, *SD* Standard deviation

**Table 4:** Multivariable linear regression model

Variable	Partial regression coefficient	95% CI (lower, upper)	Standardized β	***P***-value
Intercept	3.399	3.074, 3.724	0	< 0.0001
Ulcerative colitis, UC =1, CD = 0	0.129	0.065, 0.194	0.270	< 0.001
Droperidol concentration in the PCA solution, mcg/20 mcg of fentanyl	−0.021	− 0.033, − 0.009	−0.228	< 0.001
Intraoperative fentanyl consumption per kilogram of body weight, mcg/kg	0.023	0.009, 0.036	0.212	0.001
Age, y	−0.007	− 0.011, − 0.002	−0.201	0.004
Current smoking, yes =1, no = 0	0.111	0.025, 0.197	0.166	0.011
Diabetes mellitus, yes =1, no = 0	−0.204	−0.364, − 0.043	−0.164	0.013
Use of supplementary analgesics, yes =1, no = 0	0.066	0.007, 0.125	0.145	0.028
Administration of biologics during the month before surgery, yes =1, no = 0	0.084	0.008, 0.160	0.146	0.031
Preoperative use of opioids, yes =1, no = 0	0.066	−0.024, 0.156	0.093	0.152

*Abbreviations*: *CD* Crohn’s disease, *CI* Confidence interval, *PCA* Patient-controlled analgesia, *UC* Ulcerative colitis

### Postoperative adverse outcomes

Severe PONV, which did not improve despite the use of antiemetics, occurred in nine patients. Two of the patients who developed PONV discontinued intravenous PCA; one of them resumed intravenous PCA after effective antiemetic treatment, but the other did not resume it because of severe PONV. Two patients required temporary suspension of fentanyl administration because of severe respiratory depression. Uncontrolled pain necessitating an unscheduled intervention by a doctor was only observed in one patient. None of the patients died during the 30 days after the surgery. In the high consumption group, where the quartile range of postoperative fentanyl consumption (mcg/kg/day) was 75–100%, only one patient had severe PONV, and none showed respiratory depression or uncontrolled pain necessitating an unscheduled intervention by a doctor.

## Discussion

In this study, we showed that eight factors could predict postoperative opioid consumption in patients with IBD to some extent; these included droperidol concentration in the PCA solution, intraoperative fentanyl consumption, age, diabetes mellitus, current smoking, diagnosis of IBD (UC or CD), use of supplementary analgesics during anesthesia, and the use of biologics during the month before surgery. These factors may help clinicians optimize postoperative opioid doses to avoid insufficient pain management and complications due to opioid overdose.

To our knowledge, no previous study has sought to identify predictive factors for postoperative opioid consumption in patients with IBD. Moreover, in this study, we analyzed only routine preoperative information as candidate predictive factors, which increases the clinical applicability of our findings.

The nine variables in the regression model explained 30.2% of the variation in the response variable (R-squared = 0.302). Of these nine variables, eight showed a *P*-value of < 0.05, indicating their influence on postoperative opioid consumption.

Contrary to our expectations, none of these variables included preoperative inflammation-related factors. This may simply imply the failure of routine preoperative blood tests to distinguish the inflammatory phase from the remission phase. Elevated WBC count, CRP level, ESR, NLR and PLR in patients with IBD have been reported in previous studies; however, the discriminative value of a single biomarker in terms of disease activity has not yet been established [13–17].

Nevertheless, patients who took biologics or tumor necrosis factor (TNF) inhibitors during the month before surgery showed higher postoperative fentanyl consumption, with an approximate increase of 8.4% in estimated fentanyl consumption. The use of TNF inhibitors indicates the inflammatory phase rather than the remission phase. This may support the results of the aforementioned study by Fleyfel et al., who reported that perioperative opioid consumption in the acute inflammatory phase was higher than that in the recovery phase in the same patient [6].

Mixing droperidol in the PCA solution significantly reduced postoperative fentanyl consumption. Calculations based on the partial regression coefficient (Table 4) showed that a 1-mcg increase in droperidol concentration per 20 mcg of fentanyl decreased postoperative fentanyl consumption (mcg/kg/day) by approximately 2.1%. In most cases wherein droperidol was included in the PCA solution, approximately 10 mcg of droperidol was added per 20 mcg of fentanyl. In these cases, postoperative fentanyl consumption was expected to be reduced by approximately 21% if other factors were fixed. This might be explained by the analgesic effect of droperidol [18]. It was also used with the expectation of reducing fentanyl-induced nausea, so that the patients would not have to refrain from using fentanyl boluses despite postoperative pain. The antiemetic effect may theoretically increase opioid consumption; nonetheless, we observed a decrease in opioid consumption owing to its analgesic effect. The addition of droperidol appeared to be a useful option to reduce fentanyl dosage.

We also observed that fentanyl consumption decreased with increasing patient age. This corresponded with the findings of previous studies [7–10]. According to the regression model, a 1-year increase in age would lead to an approximately 0.7% decrease in postoperative fentanyl consumption.

Intraoperative fentanyl consumption had an increasing effect on postoperative fentanyl consumption. If other factors were fixed, a 1-mcg/kg increase in intraoperative fentanyl consumption was expected to lead to an approximate increase of 2.3% in postoperative fentanyl consumption. Considering that intraoperative fentanyl consumption is solely determined by the anesthesiologist, the anesthesiologist’s judgment that the patient is a high fentanyl consumer, regardless of whether it was judged from the changes in monitored vital signs, extent of surgery, patient background, or any other information, seems relatively correct. The same is true for the use of supplementary analgesics, such as non-steroidal anti-inflammatory drugs, acetaminophen, or pethidine. Patients who used intraoperative analgesics other than fentanyl or remifentanil had higher postoperative fentanyl consumption, with an estimated increase of approximately 6.6% in fentanyl consumption (mcg/kg/day).

Patients with diabetes mellitus showed lower postoperative fentanyl consumption, with an approximate decrease of 20.4% in estimated fentanyl consumption. A reduction in pain sensitivity is a known symptom in diabetic peripheral neuropathy. Whether the patients with diabetes included in this study had neuropathy is unclear, but this may explain the mechanism of lower fentanyl consumption in these patients. In contrast, few studies with small sample sizes have reported increased postoperative opioid requirements in patients with diabetes [19–21]. Therefore, the effect of diabetes on postoperative opioid requirement needs further investigation in larger populations.

Smoking is a risk factor for chronic pain through complex mechanisms, including interaction with opioids, altered pain processing, and psychosocial factors [22]. Studies have reported that smokers used more opioid analgesics [23, 24] and had higher pain scores [25] than did non-smokers after surgery. This tendency was also seen in the present study, with an approximate increase of 11.1% in estimated fentanyl consumption.

In this study, patients with CD used less fentanyl than did patients with UC. The partial regression coefficient (Table 4) showed that patients with UC were expected to have an approximate increase of 12.9% in fentanyl consumption than were patients with CD. These two diseases require different surgical procedures, which might lead to different intensities of pain. We could not identify any previous studies that compared opioid consumption directly between UC and CD. The differences in opioid consumption between these two diseases or individual surgical procedures warrant further investigation.

Approximately 77% (138/179) of the patients received abdominal wall nerve blocks, which included transversus abdominis plane block, quadratus lumborum block, or a combination of both blocks. All these nerve blocks were administered in a single injection prior to the surgery. Univariable analysis showed a *P*-value of 0.658, and this variable was not selected in the stepwise selection method either. Although these blocks were performed for multimodal analgesia, a single injection of ropivacaine or levobupivacaine may not have had a substantial effect on postoperative pain at 24 h. Studies have also shown the efficacy of nerve blocks to some extent, but the results have been conflicting and procedure-specific. Howle et al. conducted a systematic review and network analysis to compare different types of regional anesthesia techniques administered to patients who underwent laparotomy. They reported that compared to the control group, the group that received single-shot abdominal wall blocks showed no statistical difference in the pain score at rest at 24 h or cumulative morphine consumption at 24 h [26]. The present study findings were consistent with this result.

### Limitations

The biggest limitation of this study is that opioid consumption as a result of using a PCA does not directly express the severity of pain or the efficacy of the administered opioid. The amount of opioid used is not always equal to the amount needed. A patient may refrain from pressing the PCA button because of nausea, or conversely, may use it repeatedly owing to anxiety. Delving into these factors, as well as the dosage, is a future research prospect. However, our primary objective was to identify predictive factors from routine preoperative information.

Other limitations arise because of the retrospective nature of this study. The severity scores of IBD and cumulative steroid doses are noteworthy; unfortunately, we could not obtain sufficient information on these. Several patients with IBD also experience long courses of illness, occasionally leading to self-interruption of treatment or repeated changes of hospitals, which results in the loss of some clinical information. The severity scores of the disease were also not included in the preoperative data. As for steroids, we investigated their usage during the month before surgery. The effect of preoperative steroid use on postoperative opioid consumption was not detected in this study.

Although many patients continued to use fentanyl PCA for several days, the first 24 h were chosen as the scope of the study because the missing values increased over time. One of the limitations of this study is that the longer-term effects were not investigated.

In Japan, fentanyl is the most common postoperative analgesic for continuous intravenous administration. Furthermore, Japan has a culture in which oral opioids are not commonly used for postoperative pain control. Therefore, this study lacks generalizability to facilities that do not use fentanyl PCA for postoperative analgesia.

## Conclusions

To conclude, nine preoperative variables in the regression model explained 30.2% of the variation in postoperative fentanyl consumption. Intraoperative fentanyl consumption, current smoking, ulcerative colitis, use of supplementary analgesics, and the use of biologics during the month before surgery had a significant increasing effect on postoperative fentanyl consumption. In contrast, droperidol concentration in the PCA solution, age, and diabetes mellitus had a significant decreasing effect. Therefore, these factors should be considered when adopting postoperative administration of intravenous fentanyl PCA for patients with IBD.

## Data Availability

The datasets generated and/or analyzed during the current study are not publicly available as the protocol submitted to the IRB in advance specifies that the researchers will retain the data, but are available from the corresponding author on reasonable request.

## Abbreviations

BMI: Body mass index
CD: Crohn’s disease
CRP: C-reactive protein
ESR: Erythrocyte sedimentation rate
IBD: Inflammatory bowel disease
NLR: Neutrophil-to-lymphocyte ratio
PCA: Patient-controlled analgesia
PLR: Platelet-to-lymphocyte ratio
PONV: Postoperative nausea and vomiting
SD: Standard deviation
TNF: Tumor necrosis factor
UC: Ulcerative colitis
VIF: Variance inflation factor
WBC: White blood cell
YMCH: Yokohama Municipal Citizen’s Hospital
